# A Systematic Review and Meta-Analysis on the Safety of Antiplatelet Discontinuation Following Stent-Assisted Coil Embolization for Cerebral Aneurysms

**DOI:** 10.3390/neurosci6020034

**Published:** 2025-04-16

**Authors:** Mohammed Maan Al-Salihi, Maryam Sabah Al-Jebur, Ahmed Abd Elazim, Ram Saha, Ahmed Saleh, Farhan Siddiq, Ali Ayyad

**Affiliations:** 1 Zeenat Qureshi Stroke Institute, University of Missouri, Columbia, MO 65211, USA; 2 College of Medicine, University of Baghdad, Baghdad 00964, Iraq; 3 Department of Neurology, University of South Dakota, Sioux Falls, SD 57107, USA; 4 Department of Neurology, Virginia Commonwealth University, Richmond, VA 23284, USA; 5 Department of Neurosurgery, Hamad General Hospital, Doha 00974, Qatar; 6 Department of Neurosurgery, University of Missouri, Columbia, MO 65211, USA; 7 Department of Neurosurgery, Saarland University Hospital, 66421 Homburg, Germany

**Keywords:** antiplatelet, discontinuation, cerebral aneurysms, SACE, thromboembolism

## Abstract

Background: Stent-assisted coil embolization (SACE) is a common endovascular technique for managing intracranial aneurysms. The permanent presence of a stent inside the cerebral artery necessitates the postoperative use of antiplatelets. However, a consensus about how long to continue on it remains debated. This systematic review aims to discuss and quantify the risk of ischemic complications after antiplatelet discontinuation following SACE. Methods: PubMed, Cochrane Library, Scopus, and Web of Science (WOS) were systematically searched for studies assessing the outcomes after antiplatelet discontinuation following SACE for cerebral aneurysms. The primary outcome was the odds of ischemic complications after antiplatelet discontinuation. Using a random-effects model, the pooled event rate, along with a 95% confidence interval (CI), was calculated. The Comprehensive Meta-Analysis software (CMA) software was used for the analysis. The Newcastle–Ottawa Scale (NOS) was used for the quality assessment. Results: A total of five observational cohort studies were included in this systematic review. The studies recruited cases from 2009 and 2020, predominantly in Korea and Japan. Data from 18,425 cases obtained from four studies were analyzed. The duration of antiplatelet therapy varied widely across the included studies. Additionally, most studies reported a median follow-up of 24 months or more after antiplatelet discontinuation. We extracted and analyzed the odds of thromboembolic complications occurring within 6 to 24 months after the discontinuation of antiplatelets. The pooled rate of thromboembolism after antiplatelet discontinuation in this meta-analysis was 0.01 (95% CI: 0.006 to 0.018). Conclusion: This review demonstrates that the risk of thromboembolic complications after discontinuing antiplatelet therapy post-SACE is low. However, no strong consensus exists on the ideal duration for maintaining dual- or single-antiplatelet therapy. Further prospective studies with longer follow-ups are warranted to clarify the optimal durations needed to balance thromboembolic risk with hemorrhagic complications.

## 1. Introduction

Cerebral aneurysms stem from arterial wall dilation, often at weak points near bifurcations where hemodynamic stresses and structural vulnerabilities exist [1]. Major risk factors include aging, smoking, and hypertension [2]. They are classified based on their shape into fusiform or saccular categories, with the latter being the most common, accounting for 90% of all cerebral aneurysms [3]. The pooled prevalence of unruptured aneurysms has been estimated to be 3.2%. The majority of these aneurysms are silent and may be incidental imaging findings [4]. The primary concern arises from the potential for aneurysm rupture, leading to subarachnoid hemorrhage (SAH). Although the risk is as low as 0.25%, it is associated with high morbidity and mortality [5]. Ruptured cerebral aneurysms warrant an immediate intervention [6]. However, the decision to intervene in unruptured cerebral aneurysms depends on the assessed risk of rupture, a subject that remains a field of controversy in neurosurgery [7,8].

Stent-assisted coil embolization (SACE) is a widely accepted endovascular treatment for complex, broad-necked aneurysms. These stents are used to help prevent the coils from moving or protruding into the parent artery, ensuring a more secure placement. Due to differences in vessel anatomy and tortuosity, various stents have been developed over time to meet these specific needs [9,10]. Unlike non-stent-assisted coiling, SACE requires long-term postoperative antiplatelet therapy to ensure effective platelet inhibition in the presence of a permanent stent inside the intracranial vessels [11].

The use of antiplatelets is crucial following endovascular stent insertion to prevent stent thrombosis and thromboembolic complications, weighing the risk–benefit ratio with the hemorrhagic risks. Guidelines for coronary stenting recommend DAPT for one year, followed by lifelong SAPT. However, the optimal duration of antiplatelet therapy after cerebral artery stenting remains unclear [12,13]. In SACE, stents are placed in normal, non-atherosclerotic arteries, which lowers the risk of stent thrombosis compared to coronary stenting. However, reintervention in cerebral arteries is challenging. A thrombosed stent would be difficult to manage, making it crucial to avoid stent thrombosis. Discontinuing antiplatelet therapy after a certain period is justified to reduce bleeding risks. However, the optimal duration of therapy remains unclear. The lack of consensus in the literature highlights the need for further research on the appropriate length of treatment [14].

Some studies have examined the safety of antiplatelet discontinuation, aiming to determine the optimal duration of DAPT and SAPT for patients without comorbidities requiring ongoing antiplatelet therapy [15,16,17,18,19]. Based on the available data, many clinicians discontinue antiplatelets 3 to 36 months after SACE in clinical practice. However, this approach lacks strong supporting evidence. This systematic review aims to consolidate existing data on the safety of antiplatelet discontinuation after SACE for cerebral aneurysms to help determine the optimal duration of therapy.

## 2. Methods

This systematic review was conducted in accordance with the guidelines set by the Preferred Reporting Items for Systematic Reviews and Meta-Analyses (PRISMA) [20].

### 2.1. Search Strategy and Study Selection

A systematic literature search was carried out using PubMed, Scopus, the Cochrane Library, and the Web of Science (WOS) from their inception until 17 September 2024. The following search terms were used: (“Cerebral aneurysm” OR “Intracranial aneurysm” OR “Brain aneurysm”) AND (“Stent-assisted coiling” OR “SAC” OR “Aneurysm coil embolization” OR “Endovascular aneurysm treatment”) AND (“Antiplatelet therapy” OR “Dual antiplatelet therapy” OR “DAPT” OR “Ticlopidine” OR “Prasugrel” OR “Cangrelor” OR “Ticagrelor” OR “Antithrombotic therapy” OR “Aspirin” OR “Clopidogrel” OR “Platelet inhibition”). Retrieved records were exported to Endnote to detect and remove the duplicates. Relevant studies underwent a comprehensive full-text review to assess whether they fulfilled our pre-specified eligibility criteria. Each step was met by two independent reviewers. In the case of disagreements, a consensus with a third reviewer was taken into consideration.

### 2.2. Eligibility Criteria

We employed the PICOS framework to identify eligible studies as follows: Population: patients with cerebral aneurysms who underwent stent-assisted coil embolization (SACE); Intervention: antiplatelet discontinuation; Control: both double- and single-arm studies were eligible; Outcomes: safety outcomes, with thromboembolic complications as the primary outcome of interest; Study design: all study designs except case reports, case series, and reviews were included.

### 2.3. Data Extraction and Quality Assessment

Two independent reviewers extracted relevant data from the included studies. Information on study characteristics and baseline details, including the study design, location, recruitment period, primary outcomes, conclusions, sample size, age, gender, follow-up duration, comorbidities, aneurysm location, stent properties, and antiplatelet regimens, was extracted and tabulated. All safety outcomes were compiled into an Excel sheet. Only one outcome, the risk of thromboembolism, was consistently reported across the studies and subjected to meta-analysis. The odds of thromboembolic complications occurring within 24 months after the discontinuation of antiplatelet therapy were used for the analysis. We assessed the quality of the included studies using the Newcastle–Ottawa Scale (NOS) [21].

### 2.4. Statistical Analysis

The analysis was conducted using Comprehensive Meta-Analysis (CMA) software version 3.0 (Biostat, Englewood, NJ, USA). A random-effects model was adopted to consider the variability among the included studies. The pooled event rate, along with a 95% confidence interval (CI), was calculated. Heterogeneity was evaluated using the I^2^ statistic, with values over 50% and a *p*-value below 0.1, indicating significant heterogeneity [22].

## 3. Results

### 3.1. Search Results

**Figure 1:**
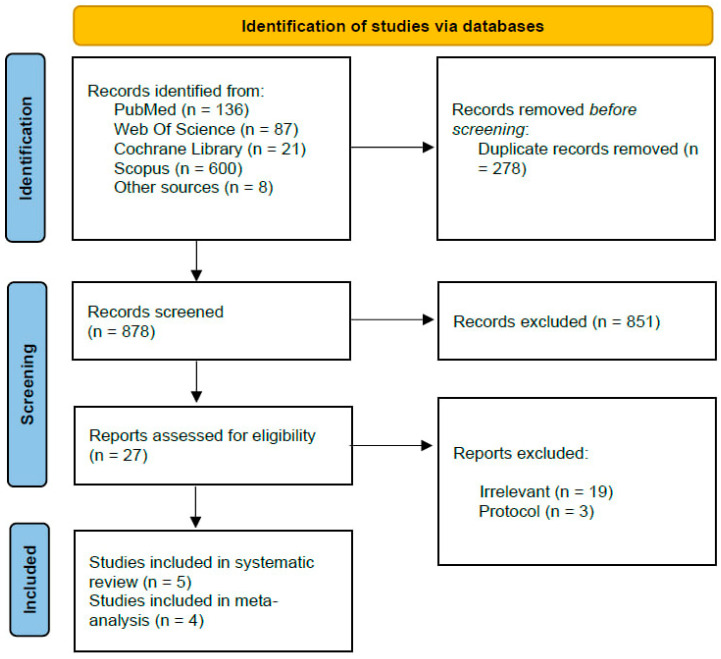
Prisma flowchart.

Our search yielded 852 records. Of these, 278 were identified as duplicates and removed. After title and abstract screening, 547 articles were found to be irrelevant. A total of 27 studies were subjected to full-text screening, of which 22 were excluded for not fully meeting the inclusion criteria. The reasons for exclusion include studies that did not focus on thromboembolic events, studies comparing DAPT vs. SAPT without discontinuation, studies not related to SACE, and study protocols. Finally, five studies met our pre-specified criteria and were included in this systematic review [15,16,17,18,19]. Reports from four out of these five studies were included in the meta-analysis [15,16,18,19]. The PRISMA flowchart is shown in Figure 1.

### 3.2. Study Characteristics and Narrative Synthesis

All studies included in this systematic review were observational cohort studies, with recruitment periods ranging from 2009 to 2020. Four studies were conducted in Korea [15,17,19], and one study was conducted in Japan [16]. Approximately 72% of the patients were females.

Baik and colleagues conducted a retrospective cohort study to explore the safety of antiplatelet discontinuation after the SACE of unruptured brain aneurysms, recruiting cases from the National Health Insurance Service (NHIS) database, which covers a large data set, reaching 17,692 patients. In this study, the authors assessed the odds of ischemic and bleeding events one month after SACE, recorded over a mean follow-up period of 4.2 years. At one year, 20.5% of patients were not on antiplatelet therapy, while 41.7% discontinued antiplatelet use two years after SACE. A time-dependent Cox proportional hazards regression model was used to evaluate the outcomes during three distinct periods following SACE: within the first year, within the second year, and after two years. However, the study did not provide detailed information regarding aneurysm location or stent types. They reported that the use of antiplatelet was associated with a 44% reduction in the risk of ischemic events in the first year after SACE, with a hazard ratio (HR) of 0.56 (95% CI 0.35 to 0.89). However, beyond the first year, this protective effect was not observed, together with an increased risk of bleeding after 2 years (HR 1.76 [95% CI 1.11 to 2.87]). After 2 years of SACE, a total number of 5147 patients were on no antiplatelets at all. Following them up for more than 24 months, only 64 patients experienced cerebral infarction (1.2%). This is the value used in our meta-analysis [19].

Hong and colleagues analyzed data from 120 patients who had antiplatelet therapy discontinued following the SACE procedure over a 10-year period. The primary outcome was thromboembolic complications related to antiplatelet discontinuation within 6 months, with lesions linked to the stented artery. Of the 120 patients, 74 (61.6%) stopped antiplatelet therapy between 18 and 36 months after SACE. During the 6-month follow-up, no patients experienced stent-related cerebral ischemia. However, transient ischemia occurred in one patient 46 months after discontinuing aspirin, though it was not related to the stented artery. This study is limited by the small sample size, which may raise concerns about potential underreporting [15].

Goto and colleagues conducted a long-term, single-center retrospective study investigating ischemic and hemorrhagic events following the discontinuation of SAPT after SACE during the period from 2010 to 2020. They included 240 patients, and the average follow-up was 46.7 months. Two years after SACE, 71.7% of patients had been taken off antiplatelets, and ischemic events were not observed at a median of 24 months, with a range from 1 to 84 months after SAPT discontinuation. They reported nine cases of ischemic complications; however, all of them occurred while the patients were on antiplatelets (SAPT or DAPT). Four out of these nine cases had Y- or T-stents, which has been reported as a significant risk factor for ischemic complications in this study group. Although ischemic events in these patients occurred while on antiplatelets, the discontinuation of antiplatelets in this subgroup of patients still warrants more caution.

Kim and colleagues conducted a multicenter retrospective study on 373 patients who had undergone SACE and discontinued antiplatelet therapy between 12 and 24 months after the procedure. DAPT maintenance durations ranged from 3 to 12 months based on different institutions. The average duration for antiplatelet use was 15.8 months, while the median clinical follow-up period after discontinuation was 24 months. No ischemic complications were observed within this follow-up, except for one high-risk patient with multiple risk factors for thromboembolism [18].

Another retrospective study involving 214 cases of patients who underwent SACE found that the discontinuation of antiplatelet therapy had no significant effect on the odds of cerebral infarction or intracranial hemorrhage. However, in terms of survival, patients who continued DAPT had better overall survival rates than those who did not. Notably, the highest mortality risk was observed in the group that stopped DAPT within 3 months after SACE, while those who continued on DAPT for 1 year had the highest survival rates. Herein, the authors recommended that DAPT should be continued for 12 months. No data were available to conclude the optimum duration of SAPT. Additionally, the study did not provide data on follow-up periods for those on or off antiplatelet therapy. Hence, this study was excluded from this meta-analysis [17]. The summary and baseline data of the included studies are shown in Table 1 and Table 2. We excluded studies that did not assess the complete discontinuation of all antiplatelet therapy (both DAPT and SAPT), as some focused only on the effects of switching from DAPT to monotherapy, which did not meet our inclusion criteria [23,24].

### 3.3. Meta-Analysis

**Figure 2:**
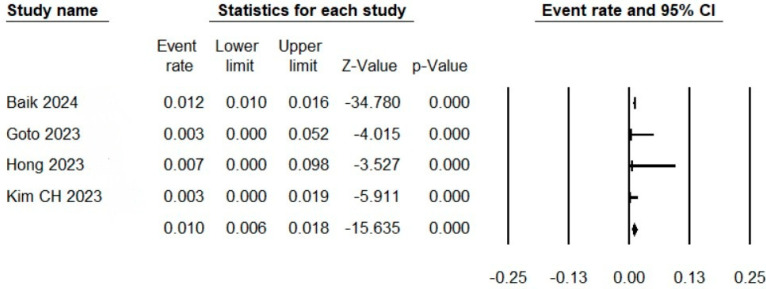
Forest plot showing the rates of thromboembolic complications after antiplatelet discontinuation.

Four studies reported the events of thromboembolism. The pooled event rate of thromboembolism after antiplatelet discontinuation in this meta-analysis was 0.01 (95% CI: 0.006 to 0.018), as shown in Figure 2.

### 3.4. Quality Assessment

The quality of each study was evaluated using the Newcastle–Ottawa Scale (NOS), which assesses the selection, comparability, and outcome. Based on this assessment, all studies were found to be of good quality (Table 3).

## 4. Discussion

In this systematic review, we summarize five studies investigating the safety outcomes after the discontinuation of antiplatelets following the SACE for cerebral aneurysms. Although the studies adopted heterogenous methodologies, the results were consistent, confirming the safety of this practice in terms of no identified risk for stent-related ischemic complications. We pooled the odds of thromboembolic complications after a follow-up of 6 to 24 months after antiplatelet discontinuation. The pooled rate in this meta-analysis was 1% (0.6% to 1.8%).

In the studies included in this systematic review, most patients discontinued antiplatelet agents 18 to 36 months after SACE. Baik and colleagues [19] reported a 41.7% discontinuation rate after 2 years, while Hong and colleagues [15] stated that 61.6% of their cases stopped antiplatelet therapy within 18 to 36 months. Goto and colleagues [16] reported the discontinuation of SAPT in 71.7% of their cohort after 24 months. Kim and colleagues [18] reported a mean duration for antiplatelet therapy of 15.8 months. One study suggested maintaining dual-antiplatelet therapy (DAPT) for at least 12 months, citing improved survival rates compared to those who received DAPT for only 3 months [17]. This recommendation aligns with the findings by Hwang et al. [23], who argued that delaying the transition from DAPT to SAPT beyond 9 months may reduce the risk of ischemic stroke. However, longer DAPT use has been associated with an increased risk of hemorrhagic events [25]. Conversely, Ozaki et al. [26] found no significant difference in the risk of ischemic stroke between patients who received 12 months of DAPT and those who had only 3 months of therapy. In another study, Ozaki et al. [27] reported no association between the duration of DAPT and the risk of ischemic stroke within 15 months post-SACE. The significant variation in the optimal duration of antiplatelet therapy across the studies highlights the lack of consensus regarding the ideal treatment duration. Some studies advocate for extended durations of up to 3 years, while others suggest that the duration should be determined based on individual patient evaluation by healthcare providers [15]. The debate is still ongoing to optimize the balance between preventing thromboembolic events and minimizing the risk of hemorrhagic complications. Baik et al. reported that in the first year, antiplatelets reduced the risk of ischemic events, with no detected difference after 2 years, together with an increased risk of bleeding [19]. Despite the variability in antiplatelet durations, discontinuing antiplatelet therapy at certain time points is a safe practice in otherwise fit patients. The pooled risk of ischemic complications in low-risk patients reported in this meta-analysis is equivalent to the estimated stroke incidence in low-risk atrial fibrillation patients who did not receive anticoagulation therapy. This translates into a low risk that does not necessitate prophylaxis as long as the patients are free from other risk factors [28].

Unlike simple coil embolization (non-stent-assisted coiling), SACE increases thrombogenicity and requires long-term postoperative antiplatelet therapy to ensure effective platelet inhibition in the presence of a permanent stent inside the intracranial vessels [11]. As a response to stent deployment inside an artery, many dynamic sequential changes occur [29].

These changes typically occur within one month, with the vessel wall reaching its maximal thickness at two months following stenting. This results in angiographically visible stent stenosis, which gradually resolves as the vessel wall becomes thinner over time. In the context of SACE, in-stent restenosis (ISR) has been reported in 2.3% to 7.8% of cases, with resolution occurring in 12.5% to 66.6% of cases within 24 months [9,30]. Based on these findings, the discontinuation of antiplatelet after 1–2 years was deemed safe in selected low-risk patients. The risk of IRS after using bare-metal stents in coronaries was reported to be 30% at 6 months [31]. The lower risks of IRS after SACE can be explained by the fact that the stents deployed in diseased stenosed arteries like coronaries expose the vessel wall to higher degrees of injury than those faced by patent cerebral vessels. This theoretically lowers the concern for stent thrombosis compared to coronary stenting. Therefore, when weighing the risks of bleeding associated with antiplatelet therapy against the potential for unnecessary lifelong use, there is a rationale for discontinuing antiplatelet treatment after a certain duration [14].

Some risk factors have been reported to be significant contributors to ischemic events after SACE. Hwang et al. reported that incomplete aneurysm occlusion after SACE was a long-term source of delayed thromboembolic complications [23]. Goto et al. reported nine cases of ischemic complications while the patients were on antiplatelet therapy (SAPT or DAPT). Four out of these nine cases had Y- or T-stents, which has been reported as a significant risk factor for delayed ischemic complications in this study group [16]. Song et al. showed that smokers and patients with aneurysms with a maximum parent artery diameter of more than 4.5 mm had more thromboembolic complications [32]. Another study by Li et al. found that a pre-SACE clinical condition and a higher dome-to-neck ratio were independent risk factors for thromboembolism [33].

The individualization of antiplatelet therapy is a critical aspect of managing patients undergoing stent implantation. Considering coronary stenting as a reference, although guidelines are well-established, clinical practice should tailor treatment to patient-specific factors. For example, in certain high-risk cases, such as patients with a higher risk of bleeding complications, DAPT may be discontinued earlier than the standard recommendation. On the other hand, some patients, particularly those with complex comorbidities, may require triple therapy (combining antiplatelets with anticoagulation), even though this regimen significantly increases the risk of bleeding complications [12,34]. Similarly, an individualized approach is essential in SACE. Based on this systematic review, low-risk patients do not require lifelong antiplatelet therapy. A duration of 12 months of DAPT may suffice, followed by 6 to 12 months of SAPT. The timing of this transition should be individualized, considering the patient’s clinical condition, comorbidities, and any adverse events during DAPT. Further studies are needed to identify the risk factors for adverse events and explore the predictive role of factors like early platelet inhibition response in guiding therapy.

We acknowledge several limitations in this systematic review and meta-analysis. All included studies were observational retrospective cohort studies, which are inherently prone to selection bias and confounding factors. Additionally, most studies were conducted in Korea and Japan, which may limit the generalizability of our findings to other populations with different genetic and lifestyle factors. The included studies exhibited variability in the durations of antiplatelet use, their definitions of thromboembolic events, and follow-up periods after discontinuation. Given that our primary aim was to systematically summarize the available evidence rather than conduct an extensive quantitative meta-analysis, we did not perform subgroup analyses for different antiplatelet regimens or durations. Each study was analyzed as a standalone data set due to the heterogeneity in study designs, and separate subgroup analyses would not have added substantial value given the limited number of studies included. Furthermore, there were no additional eligible studies that met our inclusion criteria, underscoring the need for further research in this area. Future studies should focus on larger, multicenter comparative studies with long-term follow-up, incorporating subgroup analyses based on demographic variables, stent types, comorbidities, and aneurysm characteristics. The development of risk stratification models may also enhance individualized antiplatelet management, ultimately improving patient outcomes while minimizing thromboembolic risks.

## 5. Conclusions

This systematic review demonstrates that the risk of thromboembolic complications after discontinuing antiplatelet therapy post-SACE is low. However, no strong consensus exists on the ideal duration for maintaining dual- or single-antiplatelet therapy (DAPT or SAPT). Further prospective studies with longer follow-ups are needed to clarify the optimal durations necessary to balance thromboembolic risk with hemorrhagic complications.

## Figures and Tables

**Table 1:** Summary of the included studies.

ID	Study Design	Country	Institution	Recruitment Period	Antiplatelet Regimen Used	Primary Outcomes	Conclusion
Baik 2024 [19]	Cohort	Republic of Korea	National Health Insurance Service (NHIS)	January 2009–December 2020	Aspirin, P2Y12 inhibitor, and others	The primary outcomes were cerebral infarction development and occurrence of major hemorrhage beyond 1 month following SACE.	For patients with unruptured cerebral aneurysms treated with SACE, the reasonable duration of APT for preventing cerebral infarction might be 1 year after SACE.
Goto 2023 [16]	Cohort	Japan	Nagoya University Hospital	2010–2020	Aspirin and P2Y12 inhibitor	The aim was to investigate the possibility of SAPT discontinuation and the risk factors of antiplatelet therapy reduction and termination.	It is safe to discontinue SAPT in patients without ischemic complications and with stable intraaneurysmal signals on MRA 2 years after SACE. The T- or Y-stent is a high-risk factor for delayed ischemic complications, and antiplatelet therapy reduction or discontinuation should be cautiously considered.
Hong 2023 [15]	Cohort	Republic of Korea	Seoul National University Hospital	January 2010–December 2019	Aspirin and P2Y12 inhibitor	The aim was to investigate herein the clinical outcomes experienced by patients who discontinue antiplatelet agents after SACE.	The study suggests that it may be safe to discontinue antiplatelet medication after SACE in patients at low risk for ischemia. The optimal time to discontinue might be around 18 to 36 months after SACE.
Kim TG 2023 [17]	Cohort	Republic of Korea	National Health Insurance Sharing Service	2002–2015	Aspirin and others	They aimed to find the possibility of discontinuing SAPT and to determine the proper period of DAPT use.	The continuation of antiplatelet agents or the period of DAPT use did not affect the occurrence of intracranial hemorrhage or cerebral infarction. Considering the survival rate, it would be better to maintain at least three months of antiplatelet therapy, and it might be recommended to continue DAPT use for 12 months.
Kim CH 2023 [18]	Cohort	Republic of Korea	6 Institutions	2010–2020	Aspirin and others	The aim was to investigate clinical outcomes in patients who discontinued their antiplatelet agent 12–24 months after SACE.	These results suggest that it may be safe to discontinue antiplatelet medication after SACE in patients at low risk for ischemia and that it appears safe to discontinue the agent at approximately 15 months after the procedure.

SACE; stent-assisted coil embolization, APT; antiplatelet therapy, SAPT; single-antiplatelet therapy, DAPT; dual-antiplatelet therapy, MRA; magnetic resonance angiography.

**Table 2:** Baseline characteristics of the included studies.

ID	Population (n)	Age (yrs) *	Male, n (%)	Follow-Up Period, Mos *	Comorbidity, No. (%)			Aneurysm Location, No. (%)				Stent Properties, No. (%)			
					HTN	DLD	DM	CVD	ICA	MCA	ACA	PC	Closed Cell (Laser Cut)	Closed Cell (Braided)	Open Cell
Baik 2024 [19]	17 692	57.7 ± 10.8	4169 (23.6)	50.4 ± 36	10,097 (57.07)	NA	2559 (14.46)	898 (5.05)	NA	NA	NA	NA	NA	NA	NA
Goto 2023 [16]	240	60.3 ± 12.0	84 (35.0)	46.7 ± 24.7	137 (57.1)	62 (25.8)	NA	NA	105 (43.75)	14 (5.8)	19 (7.9)	102 (42.5)	112 (46.67)	53 (22)	55 (22.91)
Hong 2023 [15]	120	56.2 ± 11.0	33 (27.5)	60.8 ± 27.9	50 (41.7)	26 (21.7)	12 (10.0)	2 (1.7)	88 (67.7)	15 (11.6)	21 (16.1)	6 (4.6)	91 (70.0)	29 (22.3)	10 (7.7)
Kim TG 2023 [17]	214	57.75 ± 12.13	NA	NA	NA	NA	NA	NA	NA	NA	NA	NA	NA	NA	NA
Kim CH 2023 [18]	373	57.8 ± 10.8	104 (27.88)	25.1 ± 16.3 (after discontinuation)	131 (35.1)	76 (20.4)	32 (8.6)	8 (2.1)	223 (59.8)	20 (5.4)	94 (25.2)	36 (9.7)	119 (30.7)	33 (8.5)	236 (60.8)

* Mean ± standard deviation, HTN; hypertension, DLD; dyslipidemia, DM; diabetes mellitus, CVD; cardiovascular disease, ACA; anterior cerebral artery, MCA; middle cerebral artery, ICA; internal carotid artery, PC; posterior circulation.

**Table 3:** Quality assessment using the NOS tool.

ID	Type of Study	Selection					Comparability	Outcome				Overall	Overall Quality
		D1	D2	D3	D4	Overall Selection		D5	D6	D7	Overall Outcome		
Kim CH 2023 [18]	Cohort	1	1	1	1	4	0	1	1	1	3	7	Good quality
Kim TG 2023 [17]	Cohort	1	1	1	1	4	1	1	1	1	3	8	Good quality
Hong 2023 [15]	Cohort	1	0	1	1	3	1	1	1	1	3	7	Good quality
Goto 2023 [16]	Cohort	1	1	1	1	4	1	1	1	1	3	8	Good quality
Baik 2024 [19]	Cohort	1	1	1	1	4	1	1	1	1	3	8	Good quality

D1: Is the case definition adequate/representative of the exposed cohort? D2: Is the case representative of the cases/selection of the non-exposed cohort? D3: Selection of controls/ascertainment of exposure. D4: Definition of controls/ demonstration that the outcome of interest was not present at the start of the study. D5: Ascertainment of exposure/assessment of outcome. D6: Was the same method of ascertainment used for cases and controls/was follow-up long enough for the outcomes to occur? D7: Non-response rate/adequacy of follow-up of cohorts.

## Data Availability

All materials related to this meta-analysis, including data collection forms, extracted data, and analysis files, will be made publicly available upon request.
